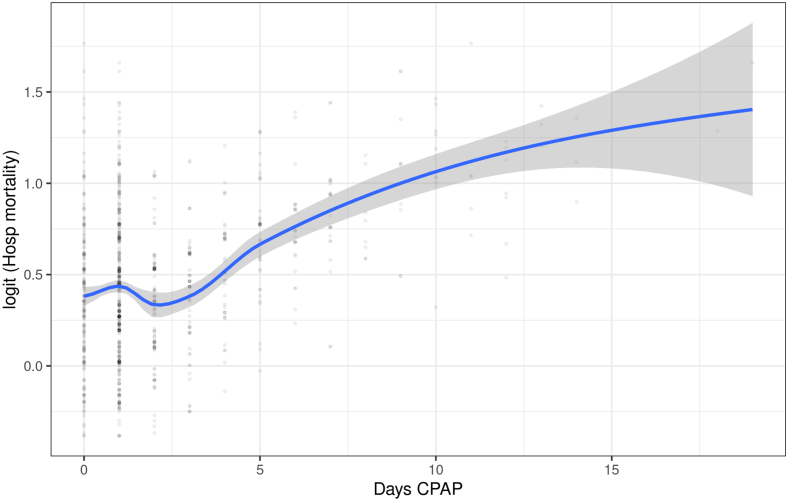# Use of protracted CPAP as supportive treatment for COVID-19 pneumonitis and associated outcomes: a national cohort study

**DOI:** 10.1016/j.bja.2023.05.012

**Published:** 2023-05-25

**Authors:** Kathryn A. Puxty, Michael Blayney, Callum Kaye, Joanne McPeake, Neil Stewart, Martin Paton, Ros Hall, Lorraine Donaldson, Nazir Lone

**Affiliations:** 1 Department of Critical Care, NHS Greater Glasgow and Clyde, Glasgow Royal Infirmary, Glasgow, UK; 2 School of Medicine, Dentistry and Nursing, University of Glasgow, Glasgow, UK; 3 Usher Institute, University of Edinburgh, Edinburgh, UK; 4 Department of Critical Care, NHS Grampian, Aberdeen Royal Infirmary, Aberdeen, UK; 5 Healthcare Improvement Scotland (Improvement Hub), Glasgow, UK; 6 The Institute of Healthcare Improvement Studies, University of Cambridge, Cambridge, UK; 7 Department of Critical Care, NHS Forth Valley, Forth Valley Royal Hospital, Larbert, UK; 8 Public Health Scotland, UK; 9 Department of Critical Care, NHS Lothian, Edinburgh Royal Infirmary, UK

**Keywords:** ARDS, continuous positive airway pressure, COVID-19, CPAP, noninvasive ventilation, pneumonitis

## Abstract

**Background:**

Continuous positive airway pressure (CPAP) has been increasingly deployed to manage patients with COVID-19 and acute respiratory failure, often for protracted periods. However, concerns about protracted CPAP have been raised. This study aimed to examine the use of CPAP for patients with COVID-19 and the outcomes after protracted use.

**Methods:**

This was a national cohort study of all adults admitted to Scottish critical care units with COVID-19 from March 1, 2020 to December 25, 2021 who received CPAP. Protracted CPAP was defined as ≥ 5 continuous days of CPAP. Outcomes included CPAP failure rate (institution of invasive mechanical ventilation [IMV] or death), mortality, and outcomes after institution of IMV. Multivariable logistic regression was performed to assess the impact of protracted CPAP on mortality after IMV.

**Results:**

A total of 1961 patients with COVID-19 received CPAP for COVID-19 pneumonitis, with 733 patients (37.4%) receiving protracted CPAP. CPAP failure occurred in 891 (45.4%): 544 patients (27.7%) received IMV and 347 patients (17.7%) died in critical care without IMV. Hospital mortality rate was 41.3% for the population. For patients who subsequently commenced IMV, hospital mortality was 58.7% for the standard duration CPAP group and 73.9% for the protracted duration CPAP group (*P*=0.003); however, there was no statistical difference in hospital mortality after adjustment for confounders (odds ratio 1.4, 95% confidence interval 0.84–2.33, *P*=0.195).

**Conclusions:**

Protracted CPAP was used frequently for managing patients with COVID-19. Whilst it was not associated with worse outcomes for those patients who subsequently required IMV, this might be due to residual confounding and differences in processes of care.

**Editor's key points**

• Face-mask CPAP is a key treatment option in acute respiratory failure.
• Many clinicians are concerned that prolonged use of CPAP could lead to worse outcomes amongst patients who then require invasive mechanical ventilation.
• This analysis did not show a significantly higher mortality rate for patients who received prolonged CPAP before invasive ventilation.
• There might still be a mortality association with prolonged CPAP but there are likely to be several causes for this, including treatment limitation decisions.
• Clinicians should reflect carefully on the next treatment steps for patients who require CPAP for more than 48 h.

Infection with SARS-CoV-2 can lead to a severe acute respiratory failure from pneumonitis, acute respiratory distress syndrome (ARDS) and pulmonary microthrombi. One in six hospitalised patients in the international severe acute respiratory infection consortium comprehensive clinical characterisation collaboration (ISARIC 4C) cohort, required admission to a critical care unit.^1^ Respiratory support varied and initial concerns regarding noninvasive ventilation (NIV) were overcome as this mode became increasingly utilised. By the second pandemic wave, the majority of patients in a critical care unit had received NIV during their stay, with continuous positive airway pressure (CPAP) the commonest mode of choice, supported by early results from RECOVERY- respiratory support (RECOVERY-RS) trial suggesting a reduction in invasive mechanical ventilation (IMV) or death with CPAP.^2^ Unlike NIV, CPAP does not provide any additional ventilatory support, but instead a set level of continuous positive pressure is applied throughout the respiratory cycle. For some patients CPAP was used as a ceiling of therapy as IMV was unlikely to be successful. Other patients were commenced on CPAP in the hope that IMV could be avoided. This resulted in some patients receiving CPAP for protracted periods in a manner not previously utilised in critical care.

Clinicians and researchers have expressed concerns about this increasing use of protracted CPAP. Evidence for ventilatory strategies in patients with ARDS has shown benefit from controlled tidal volume and pressure strategies.^3^ CPAP does not allow for tight control of these parameters, and patient self-inflicted lung injury (P-SILI), from spontaneous ventilation on CPAP, might worsen respiratory failure and subsequent outcomes for these patients.^4^ Several studies have now demonstrated higher mortality rates for patients with COVID-19 who have been treated with CPAP for more than 2–3 days compared with those receiving CPAP for shorter durations.^5^^,^^6^ However, some of these differences may be attributed to the use of CPAP as a ‘ceiling of treatment’ where the benefit of CPAP is less established.^7^ Furthermore, supportive treatment of COVID-19 has led to many patients receiving CPAP for periods far beyond that assessed in these studies. Recent national reports describe half of patients with COVID-19 managed with CPAP receive more than 5 days of support.^8^

This study describes a population with COVID-19 that received protracted CPAP (duration 5 days or more) in a critical care setting from a complete national dataset. We describe patient characteristics, baseline characteristics, tracheal intubation rate, and survival outcomes for these patients in comparison with patients who were managed with shorter-duration CPAP. In addition, the study assessed the association between protracted CPAP and outcomes after IMV for CPAP failure.

## Methods

### Study design, setting, and databases

A cohort study design was used. The Scottish Intensive Care Society Audit Group (SICSAG) database captures all adult general ICU activity within Scotland. Data are entered prospectively and are subject to regular validation assessments.^9^ The Community Health Index (CHI) number is used across Scottish health systems and uniquely identifies individuals. CHI was used to link the SICSAG database to the following national databases: Electronic Communication of Surveillance in Scotland (ECOSS) database, which captures all virology testing in Scotland; National Records of Scotland death records; and Scottish Morbidity Record 01 (SMR01), which captures all acute hospitalisations.

### Participants

The study population comprised Scottish residents aged ≥16 yr with a positive polymerase chain reaction test for nucleic acid for SARS-CoV-2 before or during critical care admission, who were admitted to general ICUs, combined ICU/high dependency units (HDUs) and standalone HDUs in Scotland, and received CPAP from March 1, 2020 to December 25, 2021. CPAP was defined as receipt of continuous positive airway pressure with or without the use of high flow nasal oxygen as required for tolerance. Method and duration of CPAP delivery was determined by the bedside clinicians. Patients who only received CPAP after extubation from IMV were excluded. Records relating to patients transferred between HDUs and ICUs were combined to create a continuous critical care stay. Only first admissions for patients with multiple, non-continuous critical care admissions were included. Follow-up was censored on January 15, 2022.

### Variables

#### Primary exposure

Receipt of type of respiratory support was recorded in the SICSAG database on a daily basis. Duration of CPAP was taken from total number of consecutive days receiving CPAP in critical care (with or without high flow nasal oxygen [HFNO] as required) before critical care discharge or initiation of IMV (whichever occurred first). A hierarchy was used so that if a patient received more than one type of respiratory support in the same day, only one was recorded (IMV > CPAP > HFNO). Protracted CPAP was defined as ≥ 5 days of receipt of CPAP, as this had been identified as the median value of all NIV modalities from the national cohort report.^8^ The relationship between time on CPAP and hospital mortality was assessed using a logit plot and the threshold for protracted CPAP reviewed. Patients receiving only HFNO were excluded.

#### Outcomes

The primary outcome was hospital mortality. Secondary outcomes included CPAP failure (death or IMV during critical care stay), critical care unit mortality, organ support during critical care stay, and duration of critical care stay. Critical care outcomes were available for those patients discharged or dead on or before January 15, 2022.

#### Other variables

Patient characteristic variables consisted of sex, age, and ethnicity. Ethnicity was derived from Scottish Census 2011 categories, aggregating low frequencies.^10^ The Scottish Index of Multiple Deprivation (SIMD Version 2020),^11^ an area-based ranking index based on postcode of residence, was used to define socioeconomic deprivation represented as quintiles. Previous health status comprised the number of emergency acute hospital admissions in the year before admission, pre-admission Clinical Frailty Score (CFS), and comorbidities. Charlson-defined comorbidities and SICSAG-defined severe comorbidities were combined as previously described.^12^^,^^13^ Acute illness variables comprised the number, type, and duration of organ systems supported (cardiovascular, respiratory, and renal support). Information pertaining to treatment limitations or clinician decision-making were not available as part of the routinely collected data.

### Statistical analysis

We used R Version 3.6.1 (R Foundation for Statistical Computing, Vienna, Austria) with the packages tidyverse, survminer, finalfit, mice and splines, to analyse data. We used a significance level of 5%, 95% confidence intervals (CIs) and two-sided *P*-values. Measures of central tendency and dispersion were presented for continuous variables. The number of admissions to Scottish critical care units determined the sample size.

Baseline characteristics and outcomes were stratified by CPAP duration status (<5 days *vs* ≥ 5 days CPAP) and outcomes and were compared using Mann-Whitney *U* and χ^2^ tests. Daily frequency of bed occupancy and organ support activity were derived from augmented care period (ACP) data and presented stratified by CPAP status.^8^^,^^12^

**Figure S1:**
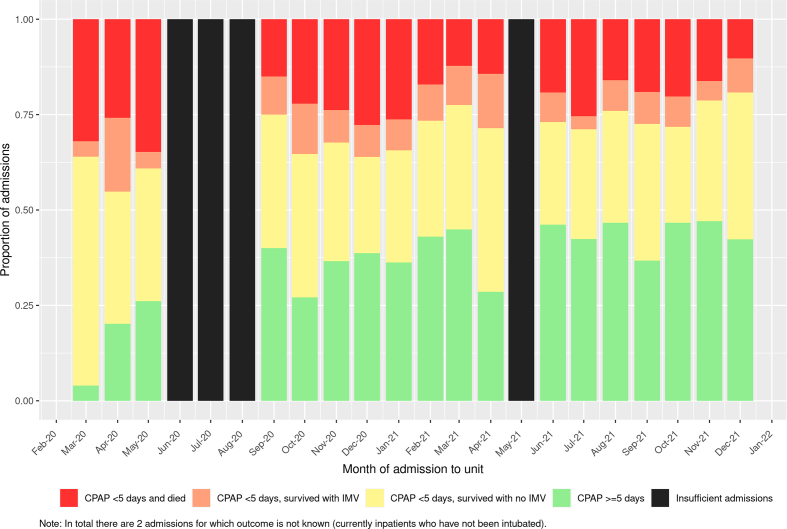


Kaplan–Meier plots were used to demonstrate mortality after institution of IMV after CPAP and groups compared with log-rank test. We evaluated the univariable and multivariable association between protracted CPAP and hospital mortality using logistic regression models, restricted to patients who transitioned from CPAP to IMV. This ensured that all patients were deemed suitable for escalation to IMV. Multiple imputation using chained equations was used to impute missing values under an assumption that data were missing at random for the following variables: frailty, SIMD, ethnicity, and hospital mortality. Five imputed datasets were created and estimates pooled using Rubin's rules.^14^ The remaining variables in the model were complete, with no missing data. Admission date was used as a measure of time. Its relationship with mortality was non-linear (Supplementary Fig. S1), likely because of changes in admission patterns and patient characteristics over time, and so the term was entered into the model as a natural spline, with five degrees of freedom. We undertook a series of sensitivity analyses, as follows. (1) We assessed the relationship between duration of CPAP before IMV (as a continuous variable) and hospital mortality. (2) We repeated analyses with a threshold of CPAP ≥ 7 days to assess the threshold of longer duration CPAP. (3) We repeated analyses in the subgroup of patients defined as non-frail using the CFS (1–3), as this group were less likely to have treatment limitations in place. (4) We repeated analyses in the subgroup of units located in hospitals in which noninvasive respiratory support for COVID-19 was provided in critical care rather than wards for the vast majority of patients (see Supplementary material) to evaluate the impact of periods of CPAP delivered in ward environments not captured in the SICSAG database.

### Approvals

The Public Benefit and Privacy Panel for Health and Social Care (1920-0093) granted SICSAG approval to undertake work relating to the COVID-19 pandemic.

## Results

Between March 1, 2020 and December 25, 2021, a total of 4829 patients with COVID-19 were admitted to Scottish critical care units. Of these, 1961 (40.6%) received CPAP as the initial ventilatory strategy (Supplementary Fig. S2); 1228 (65.7%) of those had a duration of CPAP therapy of less than 5 consecutive days (standard duration) and 733 (37.4%) had a duration of 5 or more days (protracted CPAP).

Baseline characteristics of patients who received CPAP are outlined in Table 1 and stratified according to standard or protracted duration of CPAP. All baseline features were similar between the standard and protracted CPAP groups. Median age was 61 yr and a majority (64.3%) were male; 36.4% of patients were from the most deprived SIMD quintile and 9.8% from the least deprived. A majority (95.8%) were White. Most patients (55.8%) had no co-morbidities and the most common co-morbidity was respiratory disease (17.5%). A majority of patients (69.2%) had no emergency admissions to hospital in the preceding year, 22.3% had experienced a single emergency admission, with multiple admissions uncommon (8.5%). Clinical frailty scores were available for 86.7% of the population, with the majority (56.8% overall) classified as non-frail. At presentation to critical care, partial pressure of oxygen to inspired fraction of oxygen (P:F) ratios were available for 481 (24.5%) of the total population, and 305 of these patients (63.4%) were classified as severe ARDS with P:F <13.3 kPa. Similar rates of severe ARDS were noted between the two groups (62.8% and 64.8%), although there was a higher proportion of patients with missing values in the protracted CPAP group.

**Table 1:** Characteristics of patients who received CPAP for COVID-19. CPAP indicates first set of consecutive days of CPAP before invasive ventilation, death or critical care discharge; 92 records have an unknown SIMD quintile; 186 records have unknown ethnicity. IQR, interquartile range; P:F ratio, arterial partial pressure of oxygen to inspired fraction of oxygen ratio; SIMD, Scottish Index of Multiple Deprivation. ∗P:F ratios are only available for patients admitted to level 3 or combined level 2/3 areas on the first day of their Critical Care stay and percentages given for those with data available.

		All	CPAP <5 days	CPAP ≥ 5 days
n		1961	1228	733
Age (yr)	Median (IQR)	61 (52–69)	61 (52–69.25)	61 (53–69)
Sex, n (%)	Female Male	700 (35.7) 1261 (64.3)	452 (36.8) 776 (63.2)	248 (33.8) 485 (66.2)
Socioeconomic status quintile (SIMD), n (%)	1 – Most deprived 2 3 4 5 – Least deprived	680 (36.4) 438 (23.4) 298 (15.9) 270 (14.4) 183 (9.8)	424 (36.0) 277 (23.5) 185 (15.7) 181 (15.4) 112 (9.5)	256 (37.1) 161 (23.3) 113 (16.4) 89 (12.9) 71 (10.3)
Ethnicity, n (%)	White Other	1686 (95.0%) 89 (5.0%)	1037 (94.4%) 62 (5.6%)	649 (96.0%) 27 (4.0%)
Comorbidity count, n (%)	0 1 2 plus	1095 (55.8) 414 (21.1) 452 (23.0)	685 (55.8) 265 (21.6) 278 (22.6)	410 (55.9) 149 (20.3) 174 (23.7)
Comorbidities, n (%)	Cardiovascular Respiratory Diabetes Mellitus Cancer Other	277 (14.1) 344 (17.5) 294 (15.0) 158 (8.1) 302 (15.4)	177 (14.4) 221 (18.0) 196 (16.0) 86 (7.0) 185 (15.1)	100 (13.6) 123 (16.8) 98 (13.4) 72 (9.8) 117 (16.0)
Emergency hospital admissions in previous year, n (%)	0 1 2 plus	1357 (69.2) 438 (22.3) 166 (8.5)	834 (67.9) 276 (22.5) 118 (9.6)	523 (71.) 162 (22.1) 48 (6.5)
Clinical frailty score (CFS), n (%)	Non-frail Vulnerable Frail Not known	1114 (56.8) 328 (16.%) 258 (13.2) 261 (13.3)	659 (53.7) 203 (16.5) 169 (13.8) 197 (16.0)	455 (62.1) 125 (17.1) 89 (12.1) 64 (8.7)
Time from hospital admission to critical care admission (days)	Median (IQR)	1 (0–2)	1 (0–2)	0 (0–2)
P:F ratio at admission to critical care n (%)	Median (IQR) ≤ 13.3 kPa	481 (24.5) 11.4 (9.1–15) 305 (63.4∗)	339 (27.6) 11.3 (9.0–14.8) 213 (62.8∗)	142 (19.4) 11.7 (9.4–15.3) 92 (64.8∗)

**Fig 1:**
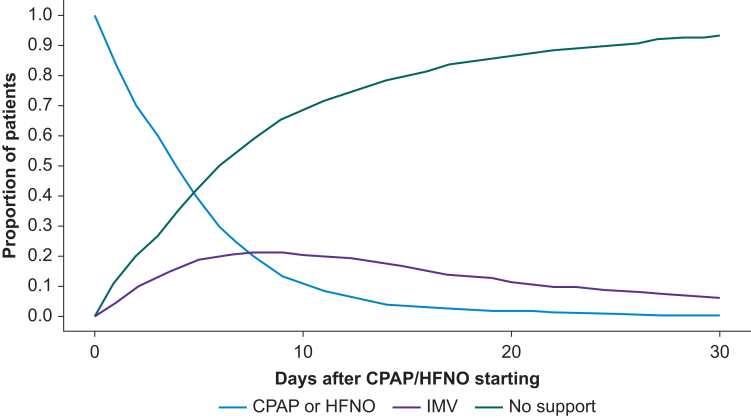
Utilisation of respiratory support for COVID-19 pneumonitis during the first 30 days after critical care admission. No support includes patients on standard facemask oxygen, nasal prongs, no oxygen, and those who have died before 30 days. HFNO, high flow nasal oxygen; IMV, invasive mechanical ventilation.

The median (interquartile range [IQR]) duration of CPAP therapy was 4 (IQR 2–7) days. The trend in use of respiratory support over the first 30 days after commencement of respiratory support is outlined in Figure 1. After initiation of CPAP, 936 patients (47.7%) also received HFNO, with more HFNO utilisation in the protracted CPAP group (60.6% *vs* 40.1%, *P*<0.001) (Table 2). There was a rapid decline in the use of CPAP over the first 10 days such that only 10% of patients who commenced CPAP remained on CPAP/HFNO at day 10.

**Table 2:** Outcomes for patients who received CPAP for COVID-19. This table includes data from 12 patients who were still in critical care at the time of the data extract. IMV, invasive mechanical ventilation; IQR, interquartile range; HFNO, high flow nasal oxygen. ∗For patients discharged alive. †Indicates data for patients who required organ support.

*n*	All	CPAP <5 days	CPAP ≥ 5 days	*P*-value
	1961	1228	733	
Outcome, *n* (%)				
Unit mortality	657 (33.6)	421 (34.3)	236 (32.4)	0.385
Hospital mortality	756 (41.3)	491 (42.8)	265 (38.8)	0.102
CPAP failure	891 (45.4)	601 (48.9)	290 (39.6)	<0.001
Required IMV	544 (27.7)	389 (31.7)	155 (21.1)	<0.001
Died in critical care without IMV	347 (17.7)	212 (17.3)	135 (18.4)	0.479
Outcome without IMV, *n* (%)				
Unit mortality without IMV	347 (24.5)	212 (25.3)	135 (23.4)	0.479
Hospital mortality without IMV	436 (33.0)	276 (35.3)	160 (29.6)	0.033
Outcome after IMV, *n* (%)				
Unit mortality after IMV	310 (57.4)	209 (53.9)	101 (66.4)	0.004
Hospital mortality after IMV	320 (63.0)	215 (58.7)	105 (73.9)	0.002
Length of stay in days, median (IQR)				
Critical care length of stay	8 (4–14)	6 (3–14)	9 (6–14)	<0.001
Post-critical care hospital stay**∗**	6 (3–14)	6 (3–13.2)	6 (3–15.2)	0.398
Total hospital length of stay	13 (8–23)	13 (7–22)	15 (10–25)	<0.001
Organ support during critical care stay, *n* (%)				
HFNO	936 (47.7)	492 (40.1)	444 (60.6)	<0.001
Cardiovascular support	592 (30.2)	411 (33.5)	181 (24.7)	<0.001
Renal support	151 (7.7)	109 (8.9)	42 (5.7)	0.012
Duration of organ support in days†, median (IQR)				
CPAP	4 (2–7)	3 (2–4)	7 (6–10)	NA
HFNO	2 (1–4)	2 (1–3)	3 (2–5)	<0.001
IMV	14 (8.8–24)	14 (9–24)	14 (8–24)	0.841
Cardiovascular support	6 (3–12)	7 (3–12)	6 (3–12)	0.663
Renal support	7 (3–14)	7 (3–15)	6 (3–13)	0.396

The proportion of patients receiving CPAP for 5 or more days was small in the first wave of the pandemic between March and May 2020. It gradually increased through the pandemic and was highest in November 2021. Trends in use of respiratory support by calendar month are outlined in Supplementary Figure S3.

### Outcomes for all patients after CPAP

Outcomes after CPAP are described in Table 2. Critical care mortality was similar between those managed with protracted CPAP and those that received a shorter CPAP course (32.4% *vs* 34.3%, *P*=0.385). Hospital mortality was also similar between the two groups at 38.8% *vs* 42.8% (*P*=0.102).

CPAP failure, defined as escalation from CPAP to IMV or death in critical care, occurred less frequently in the protracted CPAP duration group (39.6% *vs* 48.9%, *P*<0.001). This was attributable to a lower rate of IMV in the protracted duration CPAP group compared with the standard CPAP group (21.1% *vs* 31.7%, *P*<0.001). The unit mortality rate without IMV was comparable between groups (23.4% *vs* 25.3%, *P*=0.479).

Patients who received protracted CPAP were less likely to require additional organ support in the form of cardiovascular support (24.7% *vs* 33.5%, *P*<0.001) or renal replacement therapy (5.7% *vs* 8.9%, *P*=0.012). With the exception of CPAP/HFNO duration, the duration of additional organ support was similar between the two groups.

### Impact of protracted CPAP on subsequent IMV outcomes

**Fig 2:**
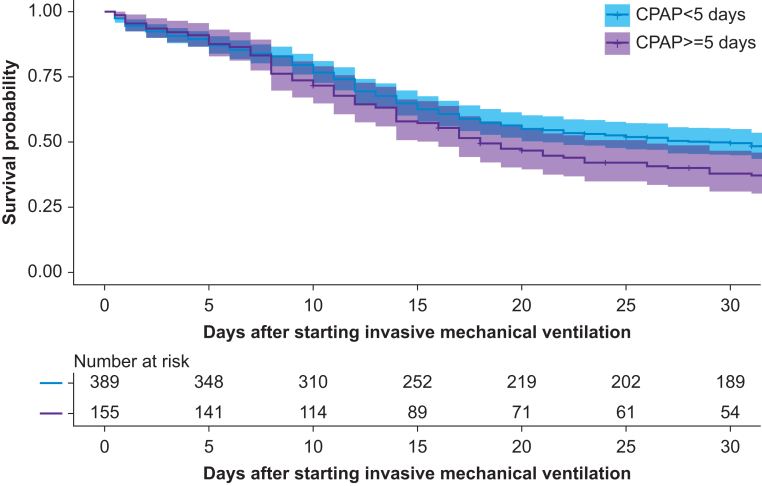
Survival after institution of IMV after CPAP for COVID-19 pneumonitis. IMV, invasive mechanical ventilation.

Baseline characteristics of patients who received IMV after a period of initial CPAP are outlined in Supplementary Table S1 and stratified according to standard or protracted duration of CPAP. The protracted CPAP group had a higher median age (62 years *vs* 59 years) and a higher proportion of males (76.1% *vs* 68.6%). Median duration of CPAP and HFNO before IMV for those with standard and protracted CPAP were: CPAP 2 days (1–3) *vs* 6 days (4–8), and HFNO 2 days (1–3) *vs* 3 days (2–5), respectively. Critical care unit and hospital mortality for patients who received IMV after CPAP was 57.4% and 63.0%, respectively (Table 2). Patients who had received protracted CPAP had higher unadjusted mortality rates when compared with standard duration CPAP; unit mortality 66.4% *vs* 53.9% (*P*=0.004) and hospital mortality 73.9% *vs* 58.7% (*P*=0.002). Kaplan–Meier survival after institution of IMV demonstrates poorer unadjusted survival for those managed with protracted CPAP (Fig. 2).

After adjusting for age, sex, deprivation, ethnicity, number of comorbidities, previous emergency admissions, frailty, P:F ratio, and ICU admission date, protracted CPAP before IMV was not associated with increased odds of hospital mortality, with an odds ratio (OR) 1.40 (95% CI 0.84–2.33, *P*=0.195) (Table 3). A sensitivity analysis using individual comorbidities did not demonstrate any difference in odds of hospital mortality (Supplementary Table S2).

**Table 3:** Factors associated with hospital mortality after invasive mechanical ventilation. Number of observations = 540. Multiple imputation has been used to supplement missing data for ultimate hospital mortality (*n*=32 [5.9 %]), SIMD (*n*=24 [4.4 %]), frailty (*n*=77 [14.3 %]) and ethnicity (*n*=40 [7.4 %]). Admission date was assessed using a natural spline with five degrees of freedom. IMV, invasive mechanical ventilation; P:F ratio, arterial partial pressure of oxygen to inspired fraction of oxygen ratio; SIMD, Scottish Index of Multiple Deprivation. ∗Age analysed as a continuous variable in years. †Includes patients with unknown ethnicity.

	Univariable model		Multivariable model	
	Odds ratio (95% CI)	*P*-value	Odds ratio (95% CI)	*P*-value
Protracted CPAP before IMV	1.94 (1.25–3.01)	0.003	1.40 (0.84–2.33)	0.195
Age∗	1.06 (1.04–1.08)	<0.001	1.07 (1.05–1.09)	<0.001
Male sex	1.59 (1.07–2.38)	0.022	1.38 (0.89–2.14)	0.155
Ethnicity (ref = White)				
Other†	0.65 (0.33–1.29)	0.218	1.11 (0.48–2.56)	0.811
SIMD (ref=5 – Least deprived)				
4	1.95 (0.5–2.23)	0.897	0.92 (0.4–2.08)	0.836
3	1.01 (0.5–2.04)	0.986	0.86 (0.39–1.89)	0.708
2	1.01 (0.51–2.02)	0.970	1.04 (0.49–2.21)	0.914
1 – Most deprived	0.82 (0.43–1.57)	0.545	0.92 (0.45–1.89)	0.828
Comorbidity (ref=none)				
Single comorbidity	0.98 (0.62–1.53)	0.914	0.97 (0.58–1.62)	0.899
Multiple comorbidities	1.47 (0.88–2.45)	0.142	1.31 (0.71–2.44)	0.387
P**:**F ratio (ref=unavailable)				
>13.3 kPa	1.09 (0.59–2.02)	0.790	1.17 (0.56–2.45)	0.674
≤ 13.3 kPa	0.99 (0.65–1.49)	0.944	0.88 (0.54–1.42)	0.595
Prior emergency admission	0.89 (0.59–1.34)	0.569	0.77 (0.48–1.25)	0.290
Frailty (ref=non-frail)				
Vulnerable or frail	0.98 (0.63–1.52)	0.925	0.8 (0.49–1.25)	0.365
Natural spline admission date				
1	2.22 (1.00–4.93)	0.051	2.27 (0.94–5.48)	0.067
2	0.58 (0.12–2.80)	0.493	1.08 (0.19–6.17)	0.932
3	1.50 (0.38–5.90)	0.558	2.20 (0.46–10.5)	0.319
4	0.88 (0.13–6.21)	0.899	1.36 (0.16–11.63)	0.780
5	7.52 (2.11–26.72)	0.002	14.98 (3.49–64.3)	<0.001

### Sensitivity analyses

The association between CPAP duration and hospital mortality varied during the first 3 days after commencement of CPAP, becoming more linear thereafter (Supplementary Fig. S4). Analyses were performed describing the population and outcomes for a protracted CPAP threshold of ≥ 7 days (Supplementary Tables S3 and S4). Outcomes were similar with an adjusted OR 1.53 (0.76–3.08, *P*=0.225) for hospital mortality after IMV after protracted CPAP of ≥ 7 days. The duration of CPAP with or without HFNO before IMV as a continuous variable was assessed after adjustment for variables (Supplementary Table S5). There was no statistically significant association between protracted CPAP (≥ 5 days) and hospital mortality in either the subgroup of non-frail patients (adjusted OR 1.32, 95% CI 0.74–2.35, *P*=0.340) (Supplementary Tables S6–S8) or the subgroup of patients receiving treatment in hospitals which provided most noninvasive respiratory support in critical care areas rather than wards (adjusted OR 1.68, 95% CI 0.88–3.20, *P*=0.117) (Supplementary Tables S9–S11).

## Discussion

One-third of patients in this national cohort study that commenced CPAP for acute respiratory failure due to COVID-19 received protracted CPAP of greater than 5 days' duration. Patient features, including baseline patient characteristics, comorbidities, and measures of frailty, were similar between those who received protracted CPAP compared with those who received CPAP for a shorter duration. One in four patients commenced on CPAP ultimately received IMV. While overall hospital mortality was similar between the two CPAP groups, protracted CPAP before IMV was associated with higher unadjusted hospital mortality. After adjusting for demographic confounders and the impact of changes in CPAP utilisation practices over time, there was no statistically significant difference in mortality noted for patients managed with protracted CPAP before IMV when compared with patients treated with a shorter duration of CPAP before IMV. This was also confirmed in subgroups of non-frail patients and those managed in hospitals which limited provision of noninvasive respiratory support to critical care units. However, these findings may be due to residual confounding and differences in processes of care between the two groups.

Previous research suggests that CPAP can be a useful strategy to manage patients with COVID-19 in need of extended respiratory support.^2^^,^^15^ Our study demonstrated that IMV was required for fewer patients managed with protracted CPAP. However, this may reflect systematic differences between the two groups, such as a lower severity of disease for those who managed to stabilise on CPAP during the initial 5 days of support. In addition, there may be a higher proportion of patients in the protracted CPAP group who were considered poor candidates for IMV. Our dataset was limited as it did not include contextual data about clinician decision-making in relation to escalation of care for individual patients. However, the baseline features between the two groups were similar in terms of age, comorbidities, and frailty. Additionally, mortality in those who did not receive IMV was similar between the two groups. There were no formal escalation policies in place in Scotland at the time of this study; however, guidelines from the British Thoracic Society and Intensive Care Society in January 2021 suggested that a lack of improvement after 3 days of CPAP management would be indicative of CPAP failure. It is unlikely that during the peak periods of COVID-19 activity that CPAP as a ceiling of treatment was continued indefinitely and, therefore, patients unsuitable for IMV may have progressed to end-of-life care before the 5 day threshold for protracted CPAP.

The RECOVERY-RS trial has provided the most robust evidence regarding noninvasive ventilation for patients with COVID-19 to date. This study randomised patients to either conventional oxygen therapy or CPAP and demonstrated a lower intubation rate in those receiving CPAP (41.3% *vs* 33.4%).^2^ Owing to randomisation, the findings of the study have a substantially lower likelihood of being affected by residual bias, in contrast to our study. However, the impact of duration of CPAP was not evaluated as part of that study.

Vaschetto and colleagues^6^ described a cohort of patients with COVID-19 treated with CPAP in respiratory intermediate care units across northern Italy. They found that more than one-third of patients commenced on CPAP outside of ICU subsequently required IMV, and that duration of CPAP therapy before IMV was associated with an increasing risk of mortality. The overall mortality rate was higher for those patients who received CPAP for greater than 3 days (51% *vs* 35%). The authors report concerns about delays in intubation leading to this increased mortality rate. While this study demonstrated similar intubation rates to our study, we did not find the same impact of protracted CPAP on outcomes after IMV. However, our study differs in that the patient population was being managed in a critical care setting with immediate IMV availability. This may have mitigated any delay to intubation when necessary.

A subsequent study conducted in 25 ICUs in Italy demonstrated that, when restricted to a population who received NIV followed by IMV, only duration of NIV administered before ICU admission and age, but not duration of NIV administered within ICU, were associated with in-hospital mortality.^5^ Our study found that hospital mortality was lower for the overall population treated with protracted CPAP compared with standard duration, but for those that required IMV, the unadjusted mortality rate was higher. This increased mortality might be expected for patients who have deteriorated in spite of in-hospital treatment and support. After adjustment for additional confounders, protracted CPAP was not associated with a statistically significantly increased odds of hospital mortality. The differences in outcomes between this study and the Italian study may be related to the differing covariates entered in multivariable models, differing patients transitioning to IMV, and differences in baseline characteristics between the study populations. Furthermore, it should be noted that all of the patients in our cohort received CPAP in a critical care setting.

This study observed a high CPAP failure rate affecting nearly half of all patients. A study reporting outcomes for 390 patients treated with NIV for COVID-19 in the health outcome predictive evaluation (HOPE) COVID-19 registry demonstrated a similar proportion with failure of noninvasive respiratory support (44%) as that seen in our cohort (45%).^16^ However, in-hospital deaths contributed 64% to this composite outcome in the HOPE study compared with 44% in our cohort. The reason for this may be attributable to differences between the cohorts, with the HOPE registry population studying all hospitalised patients rather than restricted to critical care, and having an older median age and burden of comorbidities than that observed in this study. Therefore, there have been a higher proportion of frail patients for whom IMV was deemed non-beneficial.

There are number of strengths to this study. Firstly, by linking ICU, hospitalisation records, and national death records, we are confident that we have near-complete data for the cohort of patients managed within an ICU or HDU environment in Scotland. Additionally, by linking with the ECOSS database, which records all patients with a positive COVID-19 polymerase chain reaction swab, we can be definite that all patients included had confirmed COVID-19. During the time period analysed, COVID-19 pneumonitis accounted for the vast majority of SARS-CoV-2-positive critical care admissions.

A weakness of the study is that the dataset only contains a limited amount of data regarding a patient's chronic health state and no information relating to limitations of therapy. As a result, there is a lack of granularity around why some patients were escalated to invasive ventilation or not. Furthermore, the dataset is unable to give indications about decision-making around the time of intubation and the reasoning behind a decision to intubate, therefore making it difficult to say why clinicians decided to intubate, despite a protracted period on CPAP. Factors pertaining to disease severity such as respiratory rate or respiratory effort were not available for any of the patients, and there was no measure of degree of hypoxia at the point of commencing CPAP. P:F ratios at admission to critical care were available for one-quarter of the population. These data were only collected for those patients admitted to units that provide level 3 or combined level 2/3 care and, therefore, might be expected to have a higher severity of illness. The proportion of missing data was not distributed equally, with more missing data in the protracted CPAP group. However, where data were available, the median values between the two groups were similar. P:F ratios were included in the adjusted analysis of mortality after IMV. However, with such a large proportion of missing data in the missing indicator category, the likelihood of residual confounding because of inadequate adjustment for severity of disease persists.

While the SICSAG database was adapted to respond to rapidly changing service configurations such that all areas of the hospital providing ICU level care were captured, it is recognised that some hospitals were deploying CPAP in ward environments beyond that recorded by SICSAG. It is likely that there will be a small proportion of patients who received CPAP in an external environment before being managed in a critical care unit. However, a sensitivity analysis reported similar findings when restricted to units in hospitals that limited the provision of CPAP to critical care units. Finally, the study has not assessed complications from protracted CPAP. The RECOVERY-RS trial reported the highest rate of adverse events in the CPAP group, affecting more than one-third of these participants.^2^ Moreover, poor ventilation strategies have been shown to impact longer-term outcomes in ARDS survivors.^17^

The role of NIV modalities such as CPAP in acute hypoxaemic respiratory failure is controversial, with concerns regarding uncontrolled and potentially injurious high tidal volumes exacerbating existing lung injury,^4^ delaying intubation, and leading to worse outcomes.^18^^,^^19^ Concerns have been raised during the COVID-19 pandemic regarding the role of protracted CPAP causing increased barotrauma leading to adverse outcomes on extra-corporeal ventilation.^20^ Furthermore, the increased lung stress and strain may be associated with the development or worsening of P-SILI as suggested by experts in this field.^21^ However early intubation in COVID-19 pneumonitis may have contributed to ICU strain and resource depletion during surges of pandemic activity.^22^ This study provides detailed descriptive information relating to the outcomes for patients receiving protracted CPAP in COVID-19 pneumonitis, but is limited in drawing firm conclusions relating to the relative benefits and harms of protracted CPAP compared with intubation. Whilst randomisation is the most robust approach to evaluating alternative interventions, such a study would be challenging to undertake because of logistical and ethical issues. The effects of avoiding intubation, such as less ICU-acquired weakness, dysphonia, delirium, or sedation-related complications, compared with the potential increased mental health burdens of protracted CPAP, have not been evaluated by this study and are important considerations. In addition to addressing these issues, further research should explore features such as physiological variables or patient characteristics associated with CPAP failure, in addition to exploring longer-term mortality alongside functional outcomes such as quality of life and respiratory function, to fully understand the impact of utilising protracted CPAP in this cohort.

### Conclusions

In this national cohort study of patients with COVID-19 acute respiratory failure managed with CPAP, one-third of patients received protracted CPAP. CPAP failure was common, affecting nearly half of those treated. Protracted CPAP was not associated with higher mortality rates, but this may be due to residual confounding and differences in processes of care.

## Authors’ contributions

Study conception and design: all authors

Material preparation and data collection: MP, RH, LD

Data analysis: MB

First draft of the manuscript: KP

All authors commented on previous versions of the manuscript and read and approved the final manuscript.

## Declaration of interest

The authors declare that they have no conflicts of interest. JM receives funding from THIS Institute (University of Cambridge) Research Fellowship (PD-2019-02-16). The funder had no role in the study design; collection, analysis, and interpretation of data; writing of the report; or the decision to submit the paper for publication.

## Figures and Tables

**Figure S2:**