# Supplementary figures and images for: Use of protracted CPAP as supportive treatment for COVID-19 pneumonitis and associated outcomes: a national cohort study
Source: Br J Anaesth. 2023 May 25;131(3):617–25. doi: 10.1016/j.bja.2023.05.012 (PMC10209449; doi:10.1016/j.bja.2023.05.012)

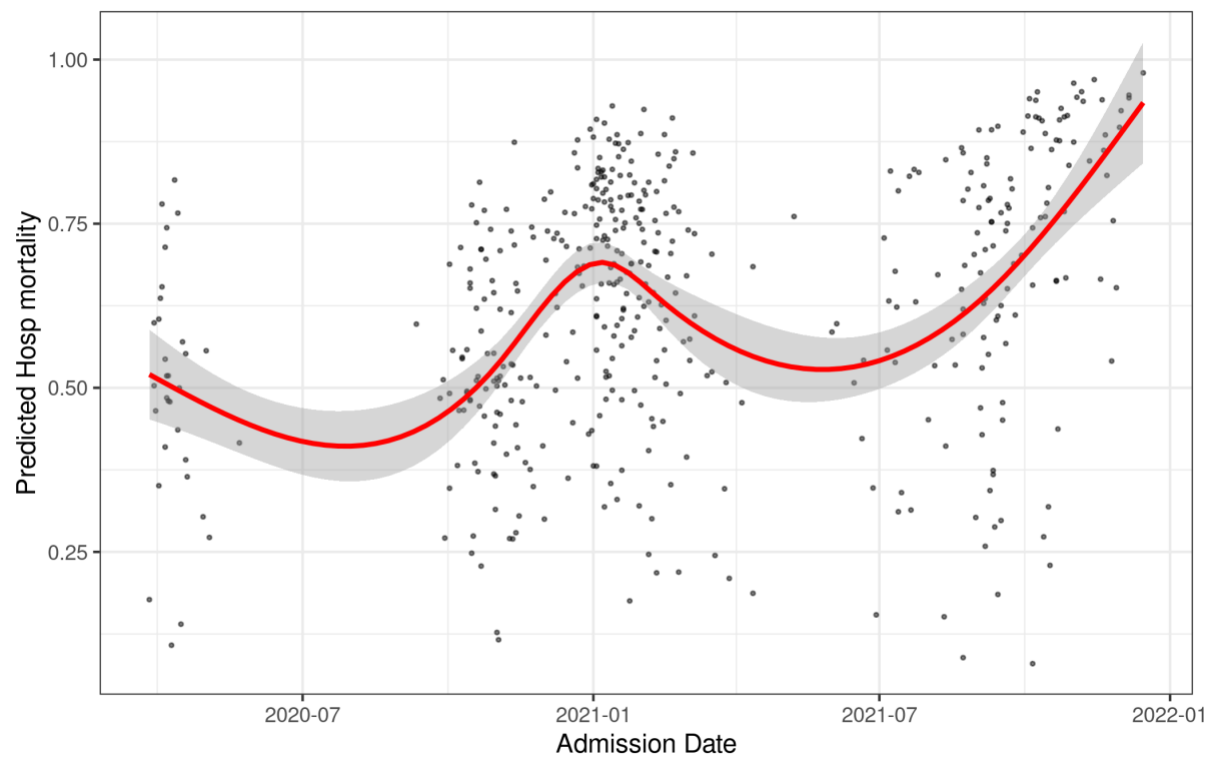

Admission date and predicted mortality

Supplement: Multimedia component 1 [file mmc1.pdf]
