# Supplementary material for: Use of protracted CPAP as supportive treatment for COVID-19 pneumonitis and associated outcomes: a national cohort study
Source: Br J Anaesth. 2023 May 25;131(3):617–25. doi: 10.1016/j.bja.2023.05.012 (PMC10209449; doi:10.1016/j.bja.2023.05.012)
Supplement: Multimedia component 2 [file mmc2.pdf]

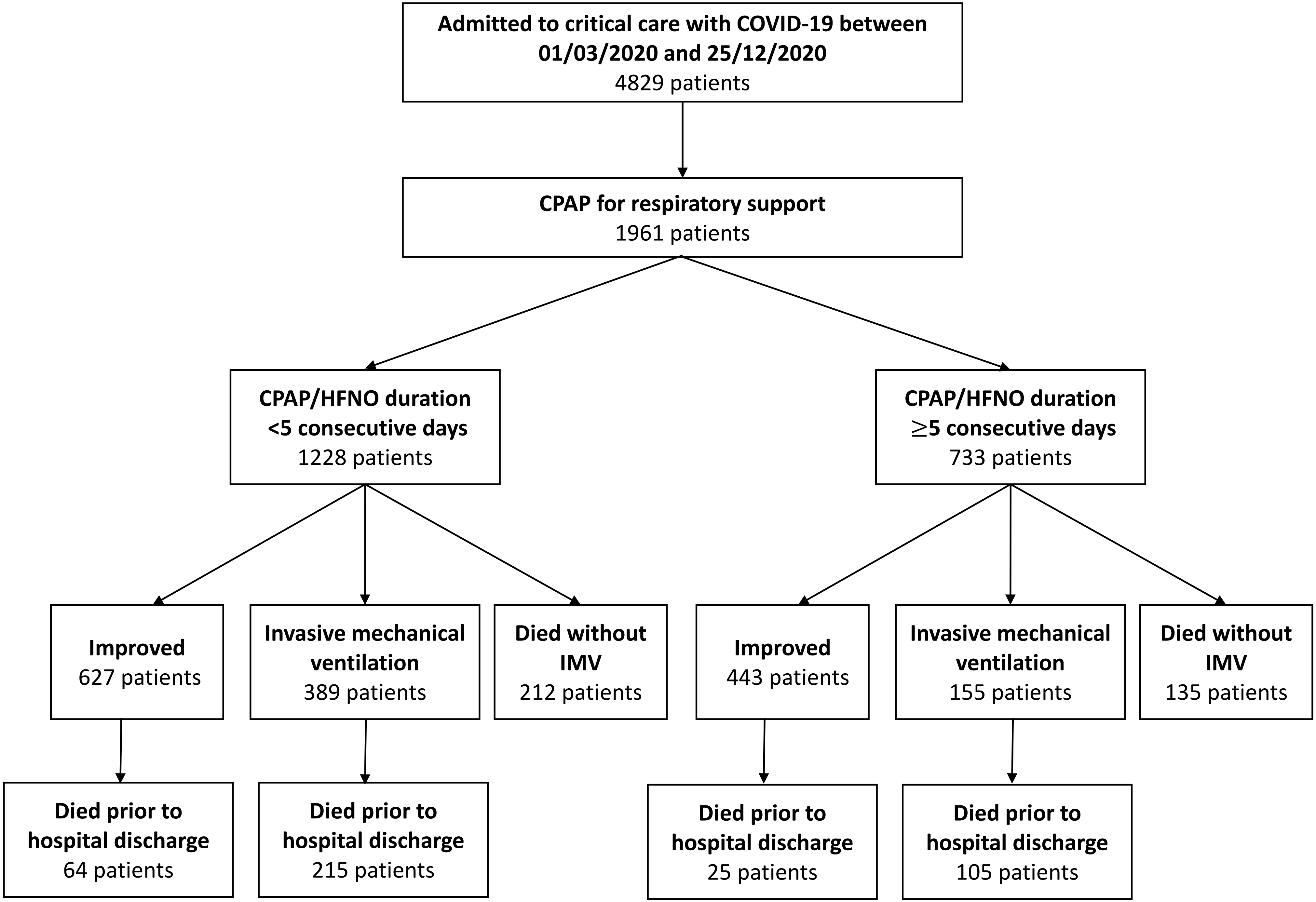
Admitted to critical care with COVID-19 between 01/03/2020 and 25/12/2020
4829 patients
CPAP for respiratory support
1961 patients
CPAP/HFNO duration <5 consecutive days
1228 patients
CPAP/HFNO duration ≥5 consecutive days
733 patients
Improved
627 patients
Invasive mechanical ventilation
389 patients
Died without IMV
212 patients
Improved
443 patients
Invasive mechanical ventilation
155 patients
Died without IMV
135 patients
Died prior to hospital discharge
64 patients
Died prior to hospital discharge
215 patients
Died prior to hospital discharge
25 patients
Died prior to hospital discharge
105 patients
