# Supplementary material for: Use of protracted CPAP as supportive treatment for COVID-19 pneumonitis and associated outcomes: a national cohort study
Source: Br J Anaesth. 2023 May 25;131(3):617–25. doi: 10.1016/j.bja.2023.05.012 (PMC10209449; doi:10.1016/j.bja.2023.05.012)
Supplement: Multimedia component 3 [file mmc3.docx]

# Supplementary material

## Supplementary tables

Supplementary Table 1: Characteristics of patients that received IMV following CPAP

Supplementary Table 2: Factors associated with hospital mortality following invasive mechanical ventilation (individual comorbidities)

Supplementary Table 3: Characteristics of patients that received IMV following CPAP comparing <7 days and ≥ 7 days CPAP

Supplementary Table 4: Factors associated with hospital mortality following invasive mechanical ventilation (individual comorbidities), protracted CPAP defined as ≥7 days

Supplementary Table 5: Factors associated with hospital mortality following invasive mechanical ventilation (Prior CPAP duration linear variable)

Supplementary Table 6: Characteristics of patients that received CPAP restricted to non-frail patients.

Supplementary Table 7: Outcomes for patients that received CPAP restricted to non-frail patients.

Supplementary Table 8: Factors associated with hospital mortality following invasive mechanical ventilation restricted to non-frail patients.

Supplementary Table 9: Characteristics of patients that received CPAP restricted to patients in hospitals in which provision of non-invasive respiratory was limited to critical care units.

Supplementary Table 10: Outcomes for patients that received CPAP restricted to patients in hospitals in which provision of non-invasive respiratory was limited to critical care units.

Supplementary Table 11: Factors associated with hospital mortality following invasive mechanical ventilation restricted to patients in hospitals in which provision of non-invasive respiratory was limited to critical care units.

## Supplementary figures

Supplementary Figure 1: Admission date and predicted mortality

Supplementary Figure 2: CPAP utilisation for COVID-19 and associated outcomes

Supplementary Figure 3: Proportions of patients managed with different modalities of respiratory support during the COVID-19 pandemic

Supplementary Figure 4: Association between CPAP duration and hospital mortality

Supplementary Table 1: Characteristics of patients that received IMV following CPAP

|  |  | | | All | CPAP <5 days | CPAP $\boldsymbol{\geq}$5 days |
| --- | --- | --- | --- | --- | --- | --- |
|  | n | | | 544 | 389 | 155 |
| Age (years) | Median (IQR) | | | 60 (52,67) | 59 (52,66) | 62 (55,69) |
| Sex | Female  Male | | | 159 (29.2%)  385 (70.8%) | 122 (31.4%)  267 (68.6%) | 37 (23.9%)  118 (76.1%) |
| Socioeconomic status quintile (SIMD) | 1 - Most deprived  2  3  4  5 - Least deprived | | | 165 (31.8%)  122 (23.5%)  90 (17.3%)  89 (17.1%)  53 (10.2%) | 120 (32.0%)  93 (24.8%)  61 (16.3%)  63 (16.8%)  38 (10.1%) | 45 (31.2%)  29 (20.1%)  29 (20.1%)  26 (18.1%)  15 (10.4%) |
| Ethnicity | White  Other | | | 468 (92.9%)  36 ( 7.1%) | 331 (92.2%)  28 ( 7.8%) | 137 (94.5%)  8 ( 5.5%) |
| Comorbidity count | 0  1  2 plus | | | 335 (61.6%)  114 (21.0%)  95 (17.5%) | 240 (61.7%)  86 (22.1%)  63 (16.2%) | 95 (61.3%)  28 (18.1%)  32 (20.6%) |
| Comorbidities | Cardiovascular  Respiratory  Diabetes Mellitus  Cancer  Other | | | 59 (10.8%)  57 (10.5%)  59 (10.8%)  39 ( 7.2%)  66 (12.1%) | 43 (11.1%)  41 (10.5%)  46 (11.8%)  22 ( 5.7%)  46 (11.8%) | 16 (10.3%)  16 (10.3%)  13 ( 8.4%)  17 (11.0%)  20 (12.9%) |
| Emergency hospital admissions in previous year | | | 0  1  2 plus | 406 (74.6%)  111 (20.4%)  27 ( 5.0%) | 293 (75.3%)  77 (19.8%)  19 ( 4.9%) | 113 (72.9%)  34 (21.9%)  8 ( 5.2%) |
| Clinical frailty score (CFS) | | Non-frail  Vulnerable  Frail  Not known | | 360 (66.2%)  67 (12.3%)  40 ( 7.4%)  77 (14.2%) | 247 (63.5%)  48 (12.3%)  30 ( 7.7%)  64 (16.5%) | 113 (72.9%)  19 (12.3%)  10 ( 6.5%)  13 ( 8.4%) |
| Time from hospital admission to critical care admission (days) Median (IQR) | | | | 0 (0,2) | 0 (0,2) | 1 (0,2) |
| Respiratory support prior to IMV  HFNO prior to IMV, n (%)  CPAP and HFNO duration, Median (IQR)  CPAP duration, Median (IQR)  HFNO duration, Median (IQR) | | | | 261 (48.0%)  4 (2,7)  2 (1,5)  2 (1,4) | 163 (41.9%)  3 (2,4)  2 (1,3)  2 (1,4) | 98 (63.2%)  7 (6,10)  6 (4.5,8)  3 (2,4) |
| P:F ratio  Available, n (%)  Median (IQR)  >13.3kPa, n (%)  $\boldsymbol{\leq}$13.3kPa, n (%) | | | | 194 (35.7%)  10.5 (8.7,13.8)  54 (27.8%)  140 (72.2%) | 163 (41.9%)  10.3 (8.6,13.7)  45 (27.6%)  118 (72.4%) | 31 (20.0%)  11.3 (8.9,14.3)  9 (29.0%)  22 (71.0%) |

CPAP indicates first set of consecutive days of CPAP prior to invasive ventilation, death or critical care discharge. 25 records have an unknown SIMD quintile. 40 records have unknown ethnicity. Percentages for organ support, advanced respiratory support, non-invasive ventilation, cardiovascular support and renal replacement therapy are based on complete recording of this data corresponding to admission date - 0 record(s) are currently missing. *Indicates cell suppression/merging due to disclosure risk. ** indicates data for only patients who required HFNO during their stay. ***PF ratio only available for patient admitted to Level 3 or Level 2/3 areas on first day of critical care stay and percentages given for those with data available.

Supplementary Table 2: Factors associated with hospital mortality following invasive mechanical ventilation (individual comorbidities)

|  | | **Univariable model** | | **Multivariable model** | |
| --- | --- | --- | --- | --- | --- |
|  | Odds ratio (95% CI) | | p-value | Odds ratio (95% CI) | p-value |
| **Protracted CPAP prior to IMV** | 1.94 (1.25,3.01) | | 0.003 | 1.44 (0.86,2.4) | 0.165 |
| **Age** | 1.06 (1.04,1.08) | | <0.001 | 1.07 (1.05,1.1) | <0.001 |
| **Male sex** | 1.59 (1.07,2.38) | | 0.022 | 1.38 (0.89,2.16) | 0.153 |
| **Ethnicity (ref= White)** | 0.65 (0.33,1.29) | | 0.218 | 1.07 (0.47,2.47) | 0.866 |
| **SIMD (ref=5 - Least deprived)** |  | |  |  |  |
| 4 | 1.95 (0.5,2.23) | | 0.897 | 0.93 (0.41,2.11) | 0.860 |
| 3 | 1.01 (0.5,2.04) | | 0.986 | 0.85 (0.39,1.86) | 0.682 |
| 2 | 1.01 (0.51,2.02) | | 0.970 | 1.03 (0.48,2.2) | 0.938 |
| 1 - Most deprived | 0.82 (0.43,1.57) | | 0.545 | 0.92 (0.45,1.89) | 0.814 |
| **Comorbidities** |  | |  |  |  |
| Cardiovascular disease | 1.54 (0.84,2.84) | | 0.161 | 1.17 (0.57,2.43) | 0.664 |
| Respiratory disease | 1.02 (0.55,1.89) | | 0.938 | 0.93 (0.46,1.9) | 0.847 |
| Diabetes Mellitus | 1 (0.57,1.76) | | 0.997 | 0.99 (0.51,1.94) | 0.979 |
| Cancer | 0.96 (0.48,1.92) | | 0.916 | 0.66 (0.3,1.44) | 0.297 |
| Other comorbidity | 1.47 (0.82,2.65) | | 0.193 | 1.44 (0.72,2.9) | 0.305 |
| **PF ratio (ref=Unavailable)** |  | |  |  |  |
| >13.3kPa | 1.09 (0.59,2.02) | | 0.790 | 1.15 (0.54,2.43) | 0.713 |
| $\leq$13.3kPa | 0.99 (0.65,1.49) | | 0.944 | 0.86 (0.53,1.39) | 0.528 |
| **Prior emergency Admission** | 0.89 (0.59,1.34) | | 0.569 | 0.8 (0.49,1.3) | 0.364 |
| **Frailty (ref= Non-frail)**  Vulnerable/Frail | 0.98 (0.63,1.52) | | 0.925 | 0.79 (0.48,1.3) | 0.350 |
| **Natural Spline Admission Date**  1 | 2.22 (1,4.93) | | 0.051 | 2.28 (0.94,5.52) | 0.067 |
| 2 | 0.58 (0.12,2.80) | | 0.493 | 1.03 (0.18,5.95) | 0.973 |
| 3 | 1.50 (0.38,5.90) | | 0.558 | 2.18 (0.44,10.76) | 0.335 |
| 4 | 0.88 (0.13,6.21) | | 0.899 | 1.36 (0.16,11.74) | 0.780 |
| 5 | 7.52 (2.11,26.72) | | 0.002 | 15.13 (3.51,65.18) | 0.000 |
| Logistic regression models showing odds ratios of various factors associated with Ultimate hospital mortality following IMV. Number of Observations = 540. Multiple imputation has been used to supplement missing data for Ultimate Hospital Mortality (n=32 (5.9 %)), SIMD (n=24 (4.4 %)), Frailty (n=77 (14.3 %)) and Ethnicity (n=40 (7.4 %). Admission date was assessed using a Natural Spline with 5 degrees of freedom. | | | | | |

Supplementary Table 3: Characteristics of patients that received IMV following CPAP comparing <7 days and $\geq$ 7 days CPAP

|  |  | | | All | CPAP <7 days | CPAP $\boldsymbol{\geq}$7 days |
| --- | --- | --- | --- | --- | --- | --- |
|  | n | | | 1961 | 1547 | 414 |
| Age (years) | Median (IQR) | | | 61 (52,69) | 61 (52,70) | 61 (53,69) |
| Sex | Female  Male | | | 700 (35.7%)  1261 (64.3%) | 543 (35.1%)  1004 (64.9%) | 157 (37.9%)  257 (39.4%) |
| Socioeconomic status quintile (SIMD) | 1 - Most deprived  2  3  4  5 - Least deprived | | | 680 (36.4%)  438 (23.4%)  298 (15.9%)  270 (14.4%)  183 ( 9.8%) | 526 (35.6%)  356 (24.1%)  235 (15.9%)  217 (14.7%)  144 ( 9.7%) | 154 (39.4%)  82 (21.0%)  63 (16.1%)  53 (13.6%)  39 (10.0%) |
| Ethnicity | White  Other | | | 468 (92.9%)  36 ( 7.1%) | 1311 (94.7%)  73 ( 5.3%) | 375 (95.9%)  16 ( 4.1%) |
| Comorbidity count | 0  1  2 plus | | | 1095 (55.8%)  414 (21.1%)  452 (23.0%) | 870 (56.2%)  331 (21.4%)  346 (22.4%) | 225 (54.3%)  83 (20.0%)  106 (25.6%) |
| Comorbidities | Cardiovascular  Respiratory  Diabetes Mellitus  Cancer  Other | | | 277 (14.1%)  344 (17.5%)  294 (15.0%)  158 ( 8.1%)  302 (15.4%) | 222 (14.4%)  268 (17.3%)  235 (15.2%)  113 ( 7.3%)  232 (15.0%) | 55 (13.3%)  76 (18.4%)  59 (14.3%)  45 (10.9%)  70 (16.9%) |
| Emergency hospital admissions in previous year | | | 0  1  2 plus | 1357 (69.2%)  438 (22.3%)  166 ( 8.5%) | 1060 (68.5%)  346 (22.4%)  141 ( 9.1%) | 297 (71.7%)  92 (22.2%)  25 ( 6.0%) |
| Clinical frailty score (CFS) | | Non-frail  Vulnerable  Frail  Not known | | 1114 (56.8%)  328 (16.7%)  258 (13.2%)  261 (13.3%) | 860 (55.6%)  248 (16.0%)  204 (13.2%)  235 (15.2%) | 254 (61.4%)  80 (19.3%)  54 (13.0%)  26 ( 6.3%) |
| Time from hospital admission to critical care admission (days) Median (IQR) | | | | 1 (0,2) | 1 (0,2) | 0 (0,2) |
| P:F ratio  Available, n (%)  Median (IQR)  >13.3kPa, n (%)  $\boldsymbol{\leq}$13.3kPa, n (%) | | | | 481 (24.5%)  11.4 (9.1,15)  176 (36.6%)  305 (63.4%) | 394 (25.5%)  11.3 (9,14.8)  143 (36.3%)  251 (63.7%) | 87 (21.0%)  12 (9.6,15.1)  33 (37.9%)  54 (62.1%) |

CPAP indicates first set of consecutive days of CPAP prior to invasive ventilation, death or critical care discharge. 92 records have an unknown SIMD quintile. 186 records have unknown ethnicity. Percentages for organ support, advanced respiratory support, non-invasive ventilation, cardiovascular support and renal replacement therapy are based on complete recording of this data corresponding to admission date - 0 record(s) are currently missing.*PF ratios are only available for patients admitted to Level 3 areas on the first day of their Critical Care stay and percentages given for those with data available.

Supplementary Table 4: Factors associated with hospital mortality following invasive mechanical ventilation (individual comorbidities), protracted CPAP defined as $\geq$7 days

|  | Univariable model | | Multivariable model | |
| --- | --- | --- | --- | --- |
|  | **OR (95% CI)** | **p-value** | **OR (95% CI)** | **p-value** |
| CPAP $\boldsymbol{\geq}$7 days prior to IMV | 2.09 (1.16,3.76) | 0.015 | 1.56 (0.8,3.06) | 0.194 |
| Age | 1.06 (1.04,1.08) | <0.001 | 1.07 (1.05,1.09) | <0.001 |
| Male sex | 1.59 (1.07,2.38) | 0.022 | 1.39 (0.9,2.17) | 0.140 |
| SIMD (ref=5 - Least deprived) |  |  |  |  |
| 4 | 1.05 (0.5,2.23) | 0.897 | 0.92 (0.4,2.09) | 0.841 |
| 3 | 1.01 (0.5,2.04) | 0.986 | 0.86 (0.39,1.88) | 0.706 |
| 2 | 1.01 (0.51,2.02) | 0.970 | 1.02 (0.48,2.18) | 0.950 |
| 1 - Most deprived | 0.82 (0.43,1.57) | 0.545 | 0.9 (0.44,1.85) | 0.778 |
| Ethnicity (ref=White)  Other | 0.65 (0.33,1.29) | 0.218 | 1.07 (0.45,2.53) | 0.874 |
| Comorbidities (ref=none) |  |  |  |  |
| Single comorbidity | 0.98 (0.62,1.53) | 0.914 | 0.97 (0.58,1.63) | 0.906 |
| Multiple comorbidities | 1.47 (0.88,2.45) | 0.142 | 1.32 (0.71,2.46) | 0.376 |
| Prior emergency admission | 0.89 (0.59,1.34) | 0.569 | 0.76 (0.47,1.23) | 0.258 |
| Frailty (ref=Non-frail)  Vulnerable or Frail | 0.98 (0.63,1.52) | 0.925 | 0.79 (0.48,1.29) | 0.342 |
| Natural Spline Admission Date 1 | 2.22 (1,4.93) | 0.051 | 2.33 (0.97,5.6) | 0.060 |
| 2 | 0.58 (0.12,2.8) | 0.493 | 1 (0.17,5.77) | 1.000 |
| 3 | 1.5 (0.38,5.9) | 0.558 | 2.33 (0.48,11.34) | 0.290 |
| 4 | 0.88 (0.13,6.21) | 0.899 | 1.26 (0.15,10.86) | 0.831 |
| 5 | 7.52 (2.11,26.72) | 0.002 | 15.16 (3.56,64.47) | <0.001 |
| PF ratio (ref=unavailable) |  |  |  |  |
| >13.3kPa | 1.09 (0.59,2.02) | 0.790 | 1.15 (0.55,2.4) | 0.715 |
| <=13.3kPa | 0.99 (0.65,1.49) | 0.944 | 0.85 (0.53,1.37) | 0.502 |

Logistic regression models showing odds ratios of various factors associated with Ultimate hospital mortality following IMV. Number of Observations = 540. Multiple imputation has been used to supplement missing data for Ultimate Hospital Mortality (n=32 (5.9 %)), SIMD (n=24 (4.4 %)), Frailty (n=77 (14.3 %)) and Ethnicity (n=40 (7.4 %). Admission date was assessed using a Natural Spline with 5 degrees of freedom

Supplementary Table 5: Factors associated with hospital mortality following invasive mechanical ventilation (Prior CPAP duration linear variable)

|  | **Univariable OR (95% CI)** | **p-value** | **Multivariable OR (95% CI)** | **p-value** |
| --- | --- | --- | --- | --- |
| **CPAP/HFNO days (prior to IMV)*** | 1.10 (1.03,1.18) | 0.006 | 1.05 (0.97,1.13) | 0.251 |
| **Age** | 1.06 (1.04,1.08) | <0.001 | 1.07 (1.05,1.09) | <0.001 |
| **Male sex** | 1.59 (1.07,2.38) | 0.022 | 1.38 (0.89,2.15) | 0.151 |
| **SIMD (ref=5 - Least deprived)** |  |  |  |  |
| 4 | 1.05 (0.5,2.23) | 0.897 | 0.92 (0.41,2.09) | 0.847 |
| 3 | 1.01 (0.5,2.04) | 0.986 | 0.86 (0.39,1.88) | 0.704 |
| 2 | 1.01 (0.51,2.02) | 0.970 | 1.03 (0.48,2.19) | 0.940 |
| 1 - Most deprived | 0.82 (0.43,1.57) | 0.545 | 0.91 (0.44,1.87) | 0.797 |
| **Ethnicity (ref=white)** Other | 0.65 (0.33,1.29) | 0.218 | 1.09 (0.47,2.55) | 0.839 |
| **Comorbidities (ref=none)** |  |  |  |  |
| Single comorbidity | 0.98 (0.62,1.53) | 0.914 | 0.96 (0.58,1.62) | 0.889 |
| Multiple comorbidities | 1.47 (0.88,2.45) | 0.142 | 1.33 (0.71,2.46) | 0.369 |
| **Prior emergency admission** | 0.89 (0.59,1.34) | 0.569 | 0.77 (0.48,1.24) | 0.281 |
| **Frailty (ref=Non-frail)** Vulnerable/Frail | 0.98 (0.63,1.52) | 0.925 | 0.78 (0.48,1.28) | 0.323 |
| **Natural Spline Admission Date** 1 | 2.22 (1,4.93) | 0.051 | 2.29 (0.95,5.53) | 0.064 |
| 2 | 0.58 (0.12,2.8) | 0.493 | 1.03 (0.18,5.94) | 0.976 |
| 3 | 1.5 (0.38,5.9) | 0.558 | 2.2 (0.46,10.57) | 0.319 |
| 4 | 0.88 (0.13,6.21) | 0.899 | 1.25 (0.14,10.78) | 0.839 |
| 5 | 7.52 (2.11,26.72) | 0.002 | 15.31 (3.6,65.21) | <0.001 |
| **PF ratio (ref=unavailable)** |  |  |  |  |
| >13.3kPa | 1.09 (0.59,2.02) | 0.790 | 1.16 (0.55,2.42) | 0.693 |
| <=13.3kPa | 0.99 (0.65,1.49) | 0.944 | 0.87 (0.54,1.41) | 0.563 |

Logistic regression models showing odds ratios of various factors associated with Ultimate hospital mortality following IMV. Number of Observations = 540. Multiple imputation has been used to supplement missing data for Ultimate Hospital Mortality (n=32 (5.9 %)), SIMD (n=24 (4.4 %)), Frailty (n=77 (14.3 %)) and Ethnicity (n=40 (7.4 %). Admission date was assessed using a Natural Spline with 5 degrees of freedom. *Duration of CPAP prior to IMV in days.

Supplementary Table 6: Characteristics of patients that received CPAP restricted to non-frail patients.

|  |  | **All** | **CPAP <5 days** | **CPAP >=5 days** |
| --- | --- | --- | --- | --- |
| Number of patients | n | 1114 | 659 | 455 |
| Age on admission (years) | Median (IQR) | 59 (50,68) | 59 (49.5,67) | 60 (51,68) |
| Sex | Female | 369 (33.1%) | 232 (35.2%) | 137 (30.1%) |
|  | Male | 745 (66.9%) | 427 (64.8%) | 318 (69.9%) |
| Socioeconomic status quintile (SIMD) | 1 - Most deprived | 374 (35.6%) | 228 (36.1%) | 146 (34.7%) |
|  | 2 | 241 (22.9%) | 143 (22.7%) | 98 (23.3%) |
|  | 3 | 167 (15.9%) | 99 (15.7%) | 68 (16.2%) |
|  | 4 | 160 (15.2%) | 102 (16.2%) | 58 (13.8%) |
|  | 5 - Least deprived | 110 (10.5%) | 59 ( 9.4%) | 51 (12.1%) |
| Ethnicity | White | 1053 (95.0%) | 622 (94.5%) | 431 (95.8%) |
|  | Black/Caribbean/  African/Other | 20 ( 1.8%) | 15 ( 2.3%) | 5 ( 1.1%) |
|  | Asian | 35 ( 3.2%) | 21 ( 3.2%) | 14 ( 3.1%) |
| **Previous health status** |  |  |  |  |
| Comorbidity count | 0 | 721 (64.7%) | 433 (65.7%) | 288 (63.3%) |
|  | 1 | 209 (18.8%) | 127 (19.3%) | 82 (18.0%) |
|  | 2 plus | 184 (16.5%) | 99 (15.0%) | 85 (18.7%) |
| Comorbidities | Cardiovascular disease | 120 (10.8%) | 69 (10.5%) | 51 (11.2%) |
|  | Respiratory disease | 127 (11.4%) | 68 (10.3%) | 59 (13.0%) |
|  | Diabetes Mellitus | 128 (11.5%) | 80 (12.1%) | 48 (10.5%) |
|  | Cancer | 74 ( 6.6%) | 38 ( 5.8%) | 36 ( 7.9%) |
|  | Other | 116 (10.4%) | 61 ( 9.3%) | 55 (12.1%) |
| Emergency hospital admissions in previous year | 0 | 845 (75.9%) | 500 (75.9%) | 345 (75.8%) |
|  | 1 | 223 (20.0%) | 130 (19.7%) | 93 (20.4%) |
|  | 2 plus | 46 ( 4.1%) | 29 ( 4.4%) | 17 ( 3.7%) |
| Clinical frailty score (CFS) | Non-frail | 1114 (100.0%) | 659 (100.0%) | 455 (100.0%) |
| **Illness severity and organ support** |  |  |  |  |
| Time from hospital admission to ICU admission (days) | Median (IQR) | 0 (0,2) | 0 (0,2) | 0 (0,2) |
| Number of organ systems supported on ICU admission | 1 | 1093 (98.1%) | 645 (97.9%) | 448 (98.5%) |
|  | 2 | 21 ( 1.9%) | 14 ( 2.1%) | 7 ( 1.5%) |
| Non-invasive respiratory support on admission | n (%) | 1114 (100.0%) | 659 (100.0%) | 455 (100.0%) |
| Cardiovascular support on admission | n (%) | 18 ( 1.6%) | 13 ( 2.0%) | 5 ( 1.1%) |
| Renal replacement therapy on admission | n (%) | 3 ( 0.3%) | 1 ( 0.2%) | 2 ( 0.4%) |
| PF ratio | Median (IQR) | 11 (8.9,14.2) | 10.6 (8.8,14.1) | 11.3 (9.1,14.8) |
|  | PF ratio Unavailable | 809 (72.6%) | 461 (70.0%) | 348 (76.5%) |
|  | >13.3 - Not Severe | 96 ( 8.6%) | 61 ( 9.3%) | 35 ( 7.7%) |
|  | <=13.3 - Severe | 209 (18.8%) | 137 (20.8%) | 72 (15.8%) |

CPAP indicates first set of consecutive days of CPAP prior to invasive ventilation, death or critical care discharge. 62 records have an unknown SIMD quintile. 6 records have unknown ethnicity. Percentages for organ support, advanced respiratory support, non-invasive ventilation, cardiovascular support and renal replacement therapy are based on complete recording of this data corresponding to admission date - 0 record(s) are currently missing. *PF ratios are only available for patients admitted to Level 3 or Level 3/2 areas on the first day of their Critical Care stay.

Supplementary Table 7: Outcomes for patients that received CPAP restricted to non-frail patients.

|  |  | **All** | **CPAP <5 days** | **CPAP >=5 days** | **p-value** |
| --- | --- | --- | --- | --- | --- |
| Number of patients | n | 1114 | 659 | 455 |  |
| **Outcome** |  |  |  |  |  |
| Unit mortality | n (%) | 325 (29.3%) | 195 (29.7%) | 130 (28.8%) | 0.759 |
| Hospital mortality | n (%) | 355 (34.5%) | 211 (34.9%) | 144 (33.9%) | 0.777 |
| Required invasive mechanical ventilation | n (%) | 360 (32.3%) | 247 (37.5%) | 113 (24.8%) | <0.001 |
| CPAP failure - invasive mechanical ventilation or died prior to discharge | n (%) | 479 (43.0%) | 307 (46.6%) | 172 (37.8%) | 0.004 |
| **Outcome without IMV** |  |  |  |  |  |
| Unit mortality without IMV | n (%) | 119 (15.8%) | 60 (14.6%) | 59 (17.3%) | 0.595 |
| Hospital Mortality without IMV | n (%) | 142 (20.4%) | 72 (19.4%) | 70 (21.7%) | 0.508 |
| **Outcome following IMV** |  |  |  |  |  |
| Unit mortality following IMV | n (%) | 206 (57.9%) | 135 (54.9%) | 71 (64.5%) | 0.040 |
| Hospital Mortality following IMV | n (%) | 213 (63.8%) | 139 (59.9%) | 74 (72.5%) | 0.037 |
| **Length of stay (days)** |  |  |  |  |  |
| Critical Care length of stay | Median (IQR) | 9 (5,16) | 8 (4,16) | 9 (6,15.5) | <0.001 |
| Post-ICU hospital stay (for patients discharged alive from ICU) | Median (IQR) | 6 (3,15) | 6 (3,14) | 6 (3,17) | 0.877 |
| Total Hospital stay | Median (IQR) | 14 (9,24) | 13 (8,23) | 15 (11,26) | <0.001 |
| **Organ support during critical care stay** |  |  |  |  |  |
| HFNO | n (%) | 596 (53.5%) | 309 (46.9%) | 287 (63.1%) | <0.001 |
| Cardiovascular support | n (%) | 376 (33.8%) | 250 (37.9%) | 126 (27.7%) | <0.001 |
| Renal support | n (%) | 86 ( 7.7%) | 57 ( 8.6%) | 29 ( 6.4%) | 0.162 |
| **Duration of organ support (days)*** |  |  |  |  |  |
| Invasive Mechanical Ventilation | Median (IQR) | 14 (8,24.2) | 14 (8.5,23.5) | 14 (8,27) | 0.845 |
| Cardiovascular support | Median (IQR) | 7 (3,12) | 7 (3,12) | 6 (2.2,12) | 0.875 |
| Renal support | Median (IQR) | 7 (3,13.8) | 8 (3,15) | 4 (3,11) | 0.320 |

Continuous Positive Airway Pressure (CPAP), Invasive Mechanical Ventilation (IMV), High Flow Nasal Oxygen (HFNO). Mortality percentages are calculated for patients who have completed their unit or hospital stay. * indicates data for patients who required organ support.

Supplementary Table 8: Factors associated with hospital mortality following invasive mechanical ventilation restricted to non-frail patients.

|  | **Univariable OR (95% CI)** | **p-value** | **Multivariable OR (95% CI)** | **p-value** |
| --- | --- | --- | --- | --- |
| CPAP >=5 days vs <5 days | 1.74 (1.05,2.89) | 0.033 | 1.32 (0.74,2.35) | 0.340 |
| Age | 1.07 (1.05,1.1) | <0.001 | 1.08 (1.05,1.11) | <0.001 |
| Male sex (ref=female) | 1.37 (0.83,2.25) | 0.218 | 1.07 (0.61,1.9) | 0.807 |
| SIMD (ref=5 - Least deprived) |  |  |  |  |
| 4 | 1.28 (0.53,3.08) | 0.578 | 1 (0.38,2.67) | 0.996 |
| 3 | 0.82 (0.34,1.95) | 0.648 | 0.6 (0.22,1.63) | 0.313 |
| 2 | 0.98 (0.42,2.29) | 0.963 | 1.04 (0.4,2.66) | 0.941 |
| 1 - Most deprived | 0.72 (0.33,1.57) | 0.402 | 0.76 (0.32,1.81) | 0.535 |
| Ethnicity - Other (ref=white) | 0.85 (0.35,2.09) | 0.729 | 1.58 (0.58,4.3) | 0.366 |
| Comorbidities (ref=none) |  |  |  |  |
| 1 comorbidity | 1.08 (0.61,1.92) | 0.782 | 1.12 (0.59,2.1) | 0.732 |
| 2 or more comorbidities | 1.93 (1.01,3.66) | 0.045 | 1.82 (0.87,3.81) | 0.113 |
| Prior emergency admission | 1.12 (0.66,1.9) | 0.668 | 0.91 (0.49,1.7) | 0.771 |
| Natural Spline Admission Date - 1 | 3.76 (0.78,18.03) | 0.098 | 5.46 (1.02,29.37) | 0.048 |
| Natural Spline Admission Date - 2 | 0.67 (0.04,10.61) | 0.773 | 3.84 (0.18,80.28) | 0.385 |
| Natural Spline Admission Date - 3 | 2.13 (0.38,11.95) | 0.389 | 3.73 (0.55,25.43) | 0.178 |
| Natural Spline Admission Date - 4 | 7.12 (0.13,398.92) | 0.338 | 49.46 (0.67,3660.59) | 0.076 |
| Natural Spline Admission Date - 5 | 2.53 (0.58,11.02) | 0.210 | 4.86 (0.95,24.94) | 0.058 |

Logistic regression models showing odds ratios of various factors associated with Ultimate hospital mortality following IMV. Number of Observations = 356. Multiple imputation has been used to supplement missing data for Ultimate Hospital Mortality (n=22 (6.2 %)), SIMD (n=15 (4.2 %)) and Ethnicity (n=1 (0.3 %). Admission date was assessed using a Natural Spline with 5 degrees of freedom.

Supplementary Table 9: Characteristics of patients that received CPAP restricted to patients in hospitals in which provision of non-invasive respiratory was limited to critical care units. Critical care audit leads were contacted in each of the 19 hospital providing continuing critical care to patients with COVID-19 during the study period. Units in 10 of the 19 hospitals provided almost all non-invasive respiratory support in critical care areas rather than ward areas. All of these units contributed data to the SICSAG audit. Analyses presented in Supplementary Table 9 to 11 are restricted to patients admitted to these 10 units.

|  |  | **All** | **CPAP <5 days** | **CPAP >=5 days** |
| --- | --- | --- | --- | --- |
| Number of patients | n | 1194 | 747 | 447 |
| Age on admission (years) | Median (IQR) | 62 (53,70) | 62 (53,71) | 62 (52.5,70) |
| Sex | Female | 429 (35.9%) | 279 (37.3%) | 150 (33.6%) |
|  | Male | 765 (64.1%) | 468 (62.7%) | 297 (66.4%) |
| Socioeconomic status quintile (SIMD) | 1 - Most deprived | 353 (31.0%) | 218 (30.5%) | 135 (31.9%) |
|  | 2 | 280 (24.6%) | 178 (24.9%) | 102 (24.1%) |
|  | 3 | 198 (17.4%) | 122 (17.1%) | 76 (18.0%) |
|  | 4 | 184 (16.2%) | 124 (17.4%) | 60 (14.2%) |
|  | 5 - Least deprived | 122 (10.7%) | 72 (10.1%) | 50 (11.8%) |
| Ethnicity | White | 1062 (97.0%) | 661 (96.6%) | 401 (97.6%) |
|  | Black/Caribbean/African/Other | 12 ( 1.1%) | 8 ( 1,1%) | 4 ( 0.9%) |
|  | Asian | 21 ( 1.9%) | 15 ( 2.2%) | 6 ( 1.5%) |
| **Previous health status** |  |  |  |  |
| Comorbidity count | 0 | 661 (55.4%) | 411 (55.0%) | 250 (55.9%) |
|  | 1 | 238 (19.9%) | 156 (20.9%) | 82 (18.3%) |
|  | 2 plus | 295 (24.7%) | 180 (24.1%) | 115 (25.7%) |
| Comorbidities | Cardiovascular disease | 178 (14.9%) | 108 (14.5%) | 70 (15.7%) |
|  | Respiratory disease | 218 (18.3%) | 134 (17.9%) | 84 (18.8%) |
|  | Diabetes Mellitus | 194 (16.2%) | 132 (17.7%) | 62 (13.9%) |
|  | Cancer | 105 ( 8.8%) | 60 ( 8.0%) | 45 (10.1%) |
|  | Other | 180 (15.1%) | 116 (15.5%) | 64 (14.3%) |
| Emergency hospital admissions in previous year | 0 | 812 (68.0%) | 491 (65.7%) | 321 (71.8%) |
|  | 1 | 268 (22.4%) | 170 (22.8%) | 98 (21.9%) |
|  | 2 plus | 114 ( 9.5%) | 86 (11.5%) | 28 ( 6.3%) |
| Clinical frailty score (CFS) | Non-frail | 655 (54.9%) | 380 (50.9%) | 275 (61.5%) |
|  | Vulnerable | 195 (16.3%) | 125 (16.7%) | 70 (15.7%) |
|  | Frail | 181 (15.2%) | 121 (16.2%) | 60 (13.4%) |
|  | Not known | 163 (13.7%) | 121 (16.2%) | 42 ( 9.4%) |
| **Illness severity and organ support** |  |  |  |  |
| Time from hospital admission to ICU admission (days) | Median (IQR) | 1 (0,2) | 1 (0,2) | 1 (0,2) |
| Number of organ systems supported on ICU admission | 1 | 1165 (97.6%) | 727 (97.3%) | 438 (98.0%) |
|  | 2 | 29 ( 2.4%) | 20 ( 2.7%) | 9 ( 2.0%) |
| Non-invasive respiratory support on admission | n (%) | 1194 (100.0%) | 747 (100.0%) | 447 (100.0%) |
| Cardiovascular support on admission | n (%) | 23 ( 1.9%) | 17 ( 2.3%) | 6 ( 1.3%) |
| Renal replacement therapy on admission | n (%) | 6 ( 0.5%) | 3 ( 0.4%) | 3 ( 0.7%) |
| PF ratio | Median (IQR) | 11.5 (9.3,15.2) | 11.3 (9.3,15.2) | 12 (9.4,15.3) |
|  | PF ratio Unavailable | 855 (71.6%) | 516 (69.1%) | 339 (75.8%) |
|  | >13.3 - Not Severe | 127 (10.6%) | 88 (11.8%) | 39 ( 8.7%) |
|  | <=13.3 - Severe | 212 (17.8%) | 143 (19.1%) | 69 (15.4%) |

Note: CPAP indicates first set of consecutive days of CPAP prior to invasive ventilation, death or critical care discharge. 57 records have an unknown SIMD quintile. 99 records have unknown ethnicity. PF ratios are only available for patients admitted to Level 3 or Level 2/3 areas on the first day of their Critical Care stay.

Supplementary Table 10: Outcomes for patients that received CPAP restricted to patients in hospitals in which provision of non-invasive respiratory was limited to critical care units.

|  |  | **All** | **CPAP <5 days** | **CPAP >=5 days** | **p-value** |
| --- | --- | --- | --- | --- | --- |
| Number of patients | n | 1194 | 747 | 447 |  |
| **Outcome** |  |  |  |  |  |
| Unit mortality | n (%) | 416 (35.0%) | 264 (35.4%) | 152 (34.4%) | 0.727 |
| Hospital mortality | n (%) | 485 (43.6%) | 315 (45.0%) | 170 (41.3%) | 0.250 |
| Required invasive mechanical ventilation | n (%) | 333 (27.9%) | 235 (31.5%) | 98 (21.9%) | <0.001 |
| CPAP failure - invasive mechanical ventilation or died prior to discharge | n (%) | 550 (46.1%) | 367 (49.1%) | 183 (40.9%) | 0.006 |
| **Outcome without IMV** |  |  |  |  |  |
| Unit mortality without IMV | n (%) | 217 (25.3%) | 132 (25.8%) | 85 (24.5%) | 0.210 |
| Hospital Mortality without IMV | n (%) | 283 (35.5%) | 181 (37.9%) | 102 (31.8%) | 0.087 |
| **Outcome following IMV** |  |  |  |  |  |
| Unit mortality following IMV | n (%) | 199 (60.5%) | 132 (56.4%) | 67 (70.5%) | 0.008 |
| Hospital Mortality following IMV | n (%) | 202 (64.3%) | 134 (60.1%) | 68 (74.7%) | 0.020 |
| **Length of stay (days)** |  |  |  |  |  |
| Critical Care length of stay | Median (IQR) | 8 (4,14) | 6 (3,13) | 9 (7,15) | <0.001 |
| Post-critical care hospital stay (for patients discharged alive from ICU) | Median (IQR) | 7 (3,15) | 7 (3,14) | 7 (3,17) | 0.246 |
| Total hospital length of stay | Median (IQR) | 14 (9,24) | 13 (7,22) | 16 (11,26) | <0.001 |
| **Organ support during critical care stay** |  |  |  |  |  |
| HFNO | n (%) | 660 (55.3%) | 331 (44.3%) | 329 (73.6%) | <0.001 |
| Cardiovascular support | n (%) | 376 (31.5%) | 251 (33.6%) | 125 (28.0%) | 0.042 |
| Renal support | n (%) | 94 ( 7.9%) | 65 ( 8.7%) | 29 ( 6.5%) | 0.169 |
| **Duration of organ support (days)*** |  |  |  |  |  |
| Advanced respiratory support | Median (IQR) | 13 (8,24) | 14 (8,24) | 13 (7.2,22.8) | 0.717 |
| Cardiovascular support | Median (IQR) | 6 (2,11) | 6 (2,11) | 5 (2,10) | 0.203 |
| Renal support | Median (IQR) | 6 (3,13) | 7 (3,14) | 4 (3,13) | 0.721 |

Continuous Positive Airway Pressure (CPAP), Invasive Mechanical Ventilation (IMV), High Flow Nasal Oxygen (HFNO). Mortality percentages are calculated for patients who have completed their unit or hospital stay. * indicates data for patients who required organ support.

Supplementary Table 11: Factors associated with hospital mortality following invasive mechanical ventilation restricted to patients in hospitals in which provision of non-invasive respiratory was limited to critical care units.

|  | **Univariable OR (95% CI)** | **p-value** | **Multivariable OR (95% CI)** | **p-value** |
| --- | --- | --- | --- | --- |
| CPAP >=5 days vs <5 days | 1.95 (1.13,3.34) | 0.016 | 1.68 (0.88,3.2) | 0.117 |
| Age | 1.06 (1.04,1.09) | <0.001 | 1.09 (1.06,1.12) | <0.001 |
| Male sex (ref=female) | 2.02 (1.22,3.35) | 0.006 | 2.14 (1.19,3.85) | 0.012 |
| SIMD (ref=5 - Least deprived) |  |  |  |  |
| 4 | 1.49 (0.6,3.68) | 0.388 | 1.33 (0.48,3.72) | 0.585 |
| 3 | 1.01 (0.4,2.52) | 0.987 | 0.79 (0.28,2.28) | 0.666 |
| 2 | 1.28 (0.56,2.93) | 0.550 | 1.42 (0.56,3.63) | 0.463 |
| 1 - Most deprived | 1.78 (0.77,4.11) | 0.174 | 2.4 (0.92,6.26) | 0.075 |
| Ethnicity - Other (ref=white) | 0.64 (0.22,1.82) | 0.397 | 0.91 (0.27,3.05) | 0.881 |
| Comorbidities (ref=none) |  |  |  |  |
| 1 comorbidity | 1.08 (0.6,1.94) | 0.790 | 0.98 (0.49,1.95) | 0.953 |
| 2 or more comorbidities | 1.18 (0.61,2.27) | 0.627 | 1.00 (0.43,2.34) | 0.999 |
| Prior emergency admission | 0.89 (0.53,1.47) | 0.638 | 0.70 (0.37,1.3) | 0.256 |
| Vulnerable/Frail (ref=non-frail) | 0.97 (0.53,1.76) | 0.916 | 0.66 (0.32,1.35) | 0.251 |
| Natural Spline Admission Date - 1 | 1.54 (0.57,4.14) | 0.396 | 1.78 (0.58,5.46) | 0.312 |
| Natural Spline Admission Date - 2 | 0.52 (0.07,3.94) | 0.527 | 1.46 (0.15,14.56) | 0.749 |
| Natural Spline Admission Date - 3 | 1.38 (0.23,8.11) | 0.723 | 2.21 (0.32,15.19) | 0.419 |
| Natural Spline Admission Date - 4 | 1.27 (0.1,16.57) | 0.855 | 2.24 (0.13,38.32) | 0.576 |
| Natural Spline Admission Date - 5 | 13.24 (2.16,81.3) | 0.006 | 34.97 (4.62,264.86) | 0.001 |

Logistic regression models showing odds ratios of various factors associated with Ultimate hospital mortality following IMV. Number of Observations = 329. Multiple imputation has been used to supplement missing data for Ultimate Hospital Mortality (n=15 (4.6 %)), SIMD (n=12 (3.6 %)), Frailty (n=55 (16.7 %)) and Ethnicity (n=23 (7 %). Admission date was assessed using a Natural Spline with 5 degrees of freedom.
